# MK2 Inhibitors as a Potential Crohn’s Disease Treatment Approach for Regulating MMP Expression, Cleavage of Checkpoint Molecules and T Cell Activity

**DOI:** 10.3390/ph15121508

**Published:** 2022-12-03

**Authors:** Eric J. Lebish, Natalie J. Morgan, John F. Valentine, Ellen J. Beswick

**Affiliations:** Division of Gastroenterology, Hepatology and Nutrition, Department of Internal Medicine, University of Utah, Salt Lake City, UT 84112, USA

**Keywords:** inflammatory bowel disease, Crohn’s Disease, MK2, MAPKAPK2, checkpoint molecules, Lag3, PD-L1, matrix metalloproteinases (MMPs)

## Abstract

Crohn’s Disease (CD) and Ulcerative Colitis (UC) are the two major forms of inflammatory bowel disease (IBD), which are incurable chronic immune-mediated diseases of the gastrointestinal tract. Both diseases present with chronic inflammation that leads to epithelial barrier dysfunction accompanied by loss of immune tolerance and inflammatory damage to the mucosa of the GI tract. Despite extensive research in the field, some of the mechanisms associated with the pathology in IBD remain elusive. Here, we identified a mechanism by which the MAPK-activated protein kinase 2 (MK2) pathway contributes to disease pathology in CD by regulating the expression of matrix metalloproteinases (MMPs), which cleave checkpoint molecules on immune cells and enhance T cell activity. By utilizing pharmaceuticals targeting MMPs and MK2, we show that the cleavage of checkpoint molecules and enhanced T cell responses may be reduced. The data presented here suggest the potential for MK2 inhibitors as a therapeutic approach for the treatment of CD.

## 1. Introduction

The two major types of inflammatory bowel disease (IBD), Crohn’s Disease (CD) and Ulcerative Colitis (UC), are incurable chronic immune-mediated diseases, but understanding the mechanisms of pathologic immune responses remains elusive. CD may affect the GI tract anywhere from the mouth to the anus, while UC is limited to the colon. These are life-long diseases that afflict over 1.6 million people in the US and have been increasing over the past decade worldwide [1]. Thus, in order to move forward in developing new effective treatment approaches, a better understanding of the mechanisms of inflammation are needed, with a focus on how CD and UC may differ in inflammatory mechanisms leading to pathogenesis of the GI tract.

One of the immune cell types that are known to be pathogenic, but are thought to differ between CD and UC, are CD4^+^ T cell responses [2]. Specifically, CD is known to have increased Th1 and Th17 responses, which may not be as prominent in UC [3]. Checkpoint molecules are key regulators of the immune response and are critical for maintaining tolerance to keep the immune system in check. In IBD, there is a loss of tolerance and overactive immune responses to normal flora and other factors in the GI tract. In a hyperactive immune state, inhibitory checkpoint molecules may be dysregulated, leading to various pathologies. While the most well-studied inhibitory checkpoint molecule on T cells is the programmed cell death protein 1 (PD-1), there are other less well studied negative regulators of T cell responses. In particular, lymphocyte-activation gene 3 (Lag3) is expressed on activated T cells and binds to Class II MHC on antigen-presenting cells [4]. Lag3 plays a known role in negatively regulating T cell activation and has also been reported to promote the inhibitory activity of regulatory T cells (Tregs) [5]. During colitis, Lag3 is thought to be expressed by regulatory T cells and can restrain gut resident macrophages and innate lymphoid cells [6]. Thus, its regulation could be an important factor in IBD, and dysregulation of this molecule could lead to increased inflammatory damage. Another inhibitory checkpoint molecule expressed on T cells is B and T lymphocyte attenuator (BTLA), which is in the B7 family of checkpoint molecules. Upon BTLA binding to its receptor, herpes virus entry mediator (HVEM), which is expressed on a variety of cell types, this interaction plays an important role in regulating T cell proliferation and cytokine production [7]. In the intestine, BTLA has been suggested to play a critical role in preventing intestinal inflammation [8].

Other inhibitory checkpoint molecules may be expressed on antigen-presenting cells, fibroblasts, and epithelial cells of the GI tract. The programmed death-ligand-1 (PD-L1) has been a major focus for various diseases for maintaining tolerance in the intestine, inhibiting T cell responses, and promoting Tregs [9]. We previously found a difference in regulation of this molecule by fibroblasts in CD vs. UC, which directly affected T cell responses. In CD, PD-L1 was downregulated in association with increased Th1- and Th17-promoting cytokines [10], which may be partially responsible for the pathogenic CD4^+^ T cell responses seen in CD. 

Matrix metalloproteinases (MMPs) are enzymes that are critical in degrading proteins in the extra cellular matrix and play a major role in tissue remodeling and repair [11,12]. In IBD, MMPs 1, 2, 3, 7, 8, 9, 10, 12, and 13 have been documented to be produced and sustained during the disease course at multiple timepoints [13,14]. In CD in particular, MMPs are known to be increased in expression and play a role in fibrosis [15,16]. However, their overall effects and potential mechanisms associated with disease are not fully understood. We previously showed that several MMPs are produced by CD-derived fibroblasts and led to decreased PD-L1 expression in fibroblast cultures [10]. In particular, MMP7, MMP9, and MMP10 were found to be produced by CD-derived fibroblasts and have effects on PD-L1 expression, leading to increased Th1 and Th17 activity. Expression of MMPs are generally thought to be regulated by tissue inhibitors of metalloproteinases (TIMPs); however, here we found a novel regulator of MMP expression by the MK2 pathway. The MK2 pathway is known for the regulation of cytokine production and plays a critical role in inflammation and cancers [17,18]. Here, we found a novel function of this pathway in regulating MMP expression in CD tissues.

For this study, we sought to uncover the underlying mechanisms of MMP production and regulation of checkpoint molecules in IBD tissues. Their overall expression in IBD tissues was examined, resulting in observed differences between CD and UC, with multiple MMPs produced at significantly higher levels in CD than in UC tissues. MMP expression was further associated with the cleavage of several critical checkpoint molecules, including Lag3, BTLA, and PD-L1, which was specific to CD. We found these processes to rely on MMP1, MMP2, and MMP12. Furthermore, we found the MK2 pathway, an important pathway in cytokine regulation and colitis [19,20], to be highly expressed and activated in CD tissues, and found that MK2 inhibitors decreased MMP expression and checkpoint molecule cleavage. Finally, we identified that MMP1, MMP2, and MMP12 can cleave Lag3 and BTLA from T cells and PD-L1 from monocytes, suggesting that MK2 is a novel regulator of MMP expression and is a potential therapeutic target for CD.

## 2. Results

### 2.1. MK2 Expression and Activity Are Increased in CD

**Figure 1:**
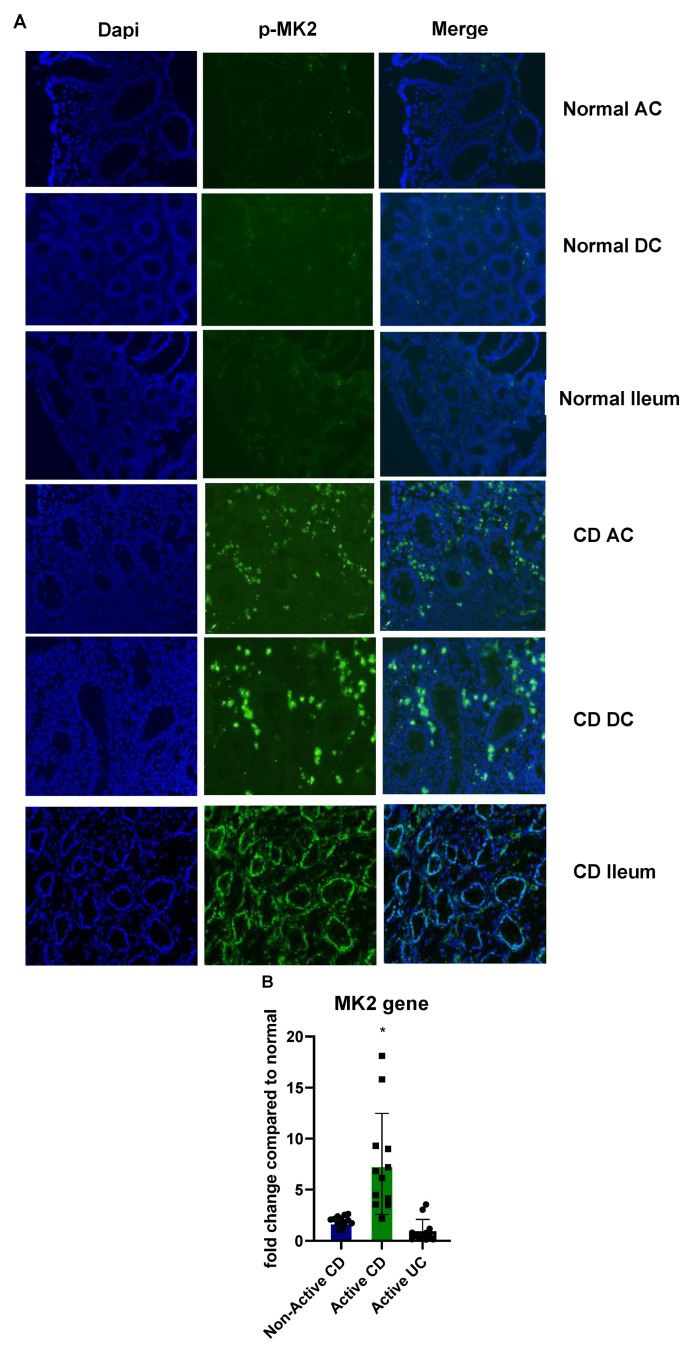
MK2 expression and activity are increased in CD tissues where (**A**) biopsies stained for p-MK2 show drastically increased staining in CD tissues compared to normal tissues and (**B**) MK2 gene expression is increased slightly in non-active CD tissues and much higher in active CD tissues, but not increased in active UC tissues. N = 12 for gene expression, * *p* < 0.05.

Because we have found the MK2 pathway to regulate inflammation, we examined its expression in CD tissues compared to normal tissues. Biopsies from patients with no known GI disease and from patients with active CD (visibly inflamed tissues) were stained for p-MK2 from multiple areas of the colon. Staining of p-MK2 was found to be very low in normal patient ascending colon (AC), descending colon (DC), and ileum, but drastically higher in active CD biopsies (Figure 1A). MK2 gene expression was also examined in panels of 12 samples where non-active CD showed a 1.78-fold increase compared to the mean of the panel of normal tissues, and active CD was found to have a mean of 7.52-fold increase (Figure 1B). UC tissues were found to have the same level of MK2 as normal tissues, suggesting that the MK2 pathway of inflammation may be specific to CD in IBD. 

### 2.2. Checkpoint Molecules Are Cleaved from CD Tissues in an MK2-Dependent Manner 

**Figure 2:**
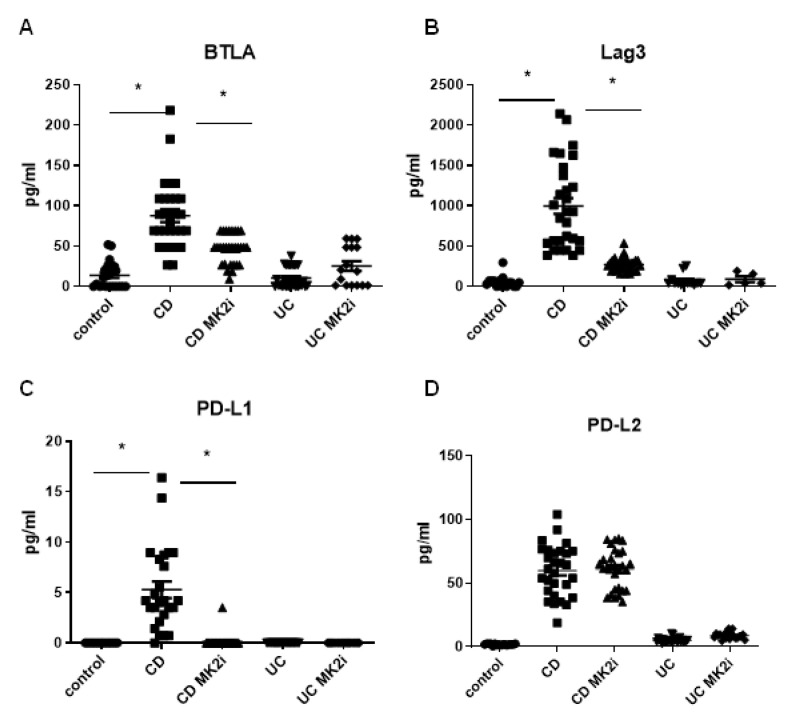
Checkpoint molecules are cleaved from CD tissues in an MK2-dependent manner where supernatants from 4 mg tissues pieces were incubated with MK2i or vehicle control and supernatants run on checkpoint multiplex array indicating that (**A**) BTLA, (**B**) Lag3, (**C**) PD-L1, and (**D**) PD-L2 were measured and shown to be cleaved into supernatants, which was reversed by MK2i treatment, but not with UC tissues. N = 23 for control, 29 for CD, and 27 for UC, * *p* < 0.05.

In IBD, there is evidence of soluble PD-L1 due to cleavage from fibroblasts [10], which led us to further examine this phenomenon. Tissues from CD and UC patients along with normal controls were divided into 4 mg sections and incubated with MK2i or vehicle control for 18 h. Supernatants were collected for analysis of checkpoint molecules by multiplex bead array. As shown in Figure 2A–D, BTLA, Lag3, PD-L1, and PD-L2 were found in supernatants in a soluble form from CD samples, but they were not significantly changed in supernatants from active UC tissues. To note, PD-1 was also examined, but not detected at significant levels in supernatants. These molecules were significantly decreased in supernatant from tissues incubated with MK2i in CD for BTLA, Lag3, and PD-L1, but not PD-L2, suggesting that MK2 may play a role in the regulation of checkpoint molecules in CD.

### 2.3. MMP Production Is Increased by CD Tissues in an MK2-Dependent Manner

**Figure 3:**
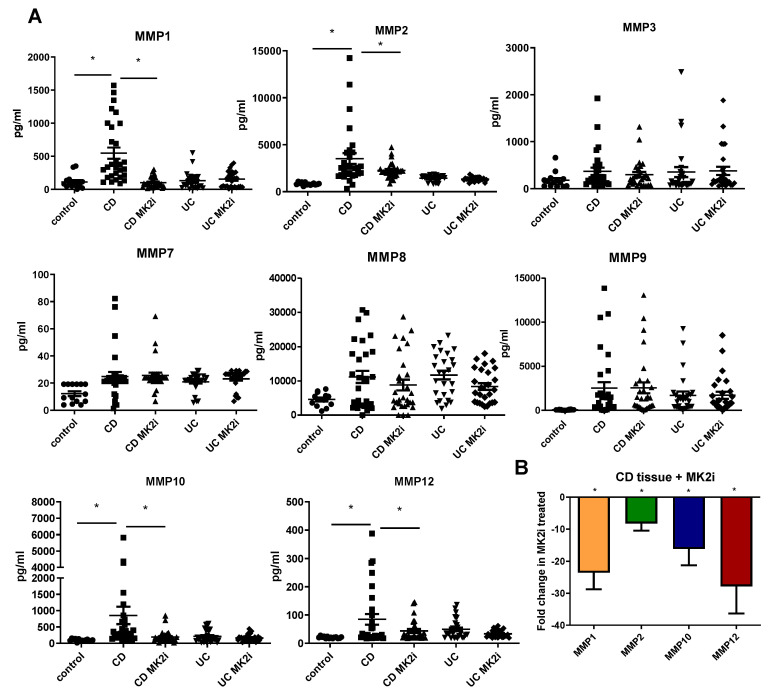
MMP production is increased in IBD tissues, but some are decreased by MK2 inhibition where (**A**) multiplex arrays of tissue supernatants indicate the MMP1, MMP2, MMP10, and MMP12 are produced by CD tissues and significantly decreased when tissues are treated with MK2 inhibitors and (**B**) gene expression of these MMP2 is also decreased by tissues incubated with MK2 inhibitors. N = 23 for control, 29 for CD, and 27 for UC, and 8 for gene expression * *p* < 0.05.

Previous work has suggested that MMPs are able to cleave checkpoint molecules [10,21], so we sought to investigate a potential link between MMPs and MK2. Supernatants from normal, CD, and UC tissues mentioned above were also examined for MMP production by multiplex bead array. MMPs were found to be produced by both CD and UC tissues at higher levels than normal tissues (Figure 3A), with MMP1, MMP2, MMP10, and MMP12 produced at significantly higher levels in CD tissues than in UC tissues. These MMPs were decreased in the supernatants of samples that had been incubated with MK2 inhibitors compared to control. At the gene level, CD samples incubated with MK2i also showed decreased gene expression of these MMPs compared to control: MMP1 at 23-fold, MMP2 at 8-fold, MMP10 at 16-fold, and MMP12 at 28-fold (Figure 3B). Thus, taken together, we have demonstrated there is an MMP expression difference between CD and UC tissues, which may be directly regulated by MK2 activity.

### 2.4. Checkpoint Molecules Are Cleaved from CD Tissues in an MMP-Dependent Manner

**Figure 4:**
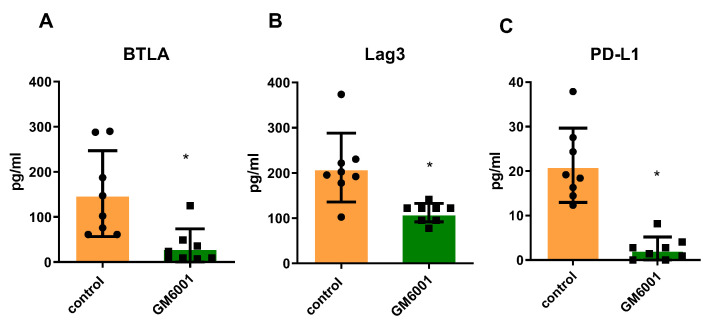
Checkpoint molecule cleavage from CD tissues is inhibited by GM6001 MMP inhibitor where the multiplex array of tissue supernatants indicates that (**A**) BTLA, (**B**) Lag2, and (**C**) PD-L1 are decreased when tissues are treated with GM6001. N = 8, * *p* < 0.05.

To investigate the direct role of MMPs in cleaving checkpoint molecules, a panel of CD tissues were incubated with the MMP inhibitor drug GM6001, in a similar approach as MK2 inhibitor described above and compared to the control. Supernatants were collected for analysis of checkpoint molecules. BTLA, Lag3, and PD-L1 were all found to be drastically decreased in the supernatants of tissues treated with GM6001 (Figure 4A–C), indicating that these molecules are cleaved in an MMP-dependent manner from CD tissues.

### 2.5. Recombinant MMPs Cleave Checkpoint Molecules from T Cells and Monocytes

**Figure 5:**
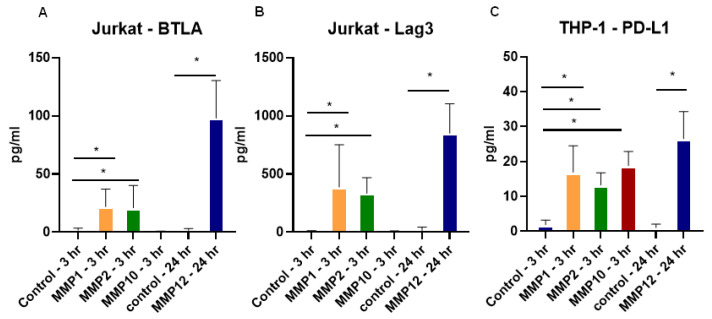
MMPs cleave checkpoint molecules from T cells and monocytes where cells are treated with recombinant MMPs activated in AMPA buffer and (**A**) BTLA and (**B**) Lag3 are cleaved from Jurkat T cell line, and (**C**) PD-L1 is cleaved from THP-1 cells in supernatants. N = 6, * *p* < 0.05.

In order to model the cell types involved in the pathogenesis of CD, Jurkat and THP-1 cells were utilized to examine the specific effect of the MMPs found in CD tissues, as shown in Figure 3A. Recombinant MMPs were incubated in an APMA-containing buffer for activation as previously described [21] and added to Jurkat cells treated with a cell stimulation cocktail for 24 h or THP-1 activated with LPS for 24 h to maximize checkpoint molecule surface expression. MMPs were added to cultures for 3 h for MMP1, MMP2, and MMP10 and 24 h for MMP12. Supernatants were analyzed for BTLA and Lag3 for Jurkat cells where MMP1, MMP2, and MMP12 were found to cleave these molecules (Figure 5A,B), but not MMP10. For THP-1 cells, PD-L1 was significantly increased in the supernatants by all four MMPs tested, which are specific to CD in our studies (Figure 5C). Taken together, these data show that MMPs have checkpoint cleavage capabilities on various immune cell types.

### 2.6. T Cell Activation and Cytokine Production Are Dependent on MMPs

**Figure 6:**
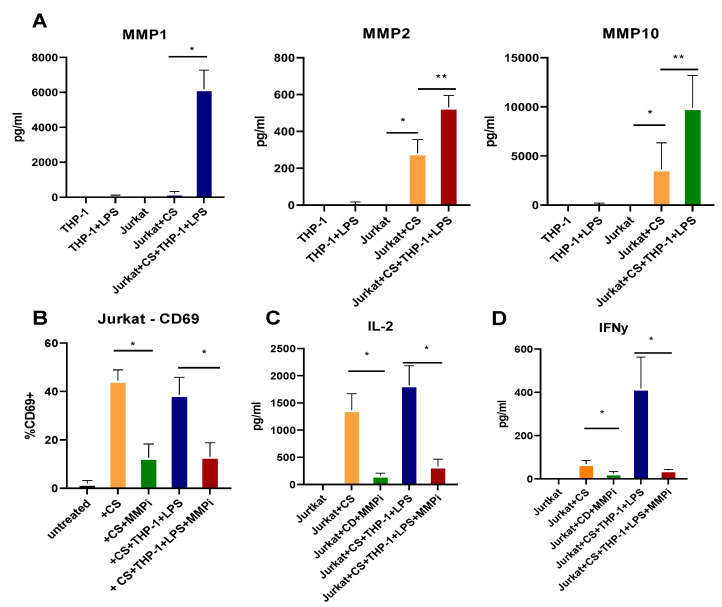
Activated T cells and T cells in co-culture with monocytes produce MMPs and are activated, which are decreased by GM6001 MMP inhibitor where (**A**) MMPs are produced and activated by Jurkat cells activated with a cell stimulation cocktail (CS) or activated in co-culture with THP-1 cells activated with LPS and (**B**) express CD69 as an activation marker that is decreased by cells treated with MMPi, as are (**C**) IL-2 and (**D**) IFNγ production. N = 6, * and ** *p* < 0.05.

As checkpoint molecules are critical in T cell activation, proliferation, and cytokine production, the impact of MMPs on Jurkat cell activity was investigated. Jurkat cells were activated with a cell stimulation cocktail, and THP-1 cells were activated with LPS for 24 h. Supernatants from each cell type and co-cultures were examined for MMPs by multiplex array. MMP1, MMP2, and MMP10 (but not MMP12) were produced in cultures, with Jurkat cells treated with cell stimulation cocktail produced MMP2 and MMP10, but MMP1 required co-culture with THP-1 cells (Figure 6A). Furthermore, these cells were stained for CD69 as a marker of activation and found to express CD69 alone and in co-culture with THP-1 cells, which was decreased when exposed to the MMP inhibitor (Figure 6B). The supernatants from these cultures also showed increased IL-2 and IFNγ, which were significantly decreased in cultures with MMP inhibitors (Figure 6C,D). These data suggest an important role for MMPs in the regulation of T cell responses that are important in CD.

### 2.7. CD Tissues Treated with MK2i or MMPi Have Decreased T Cell Activation Markers

**Figure 7:**
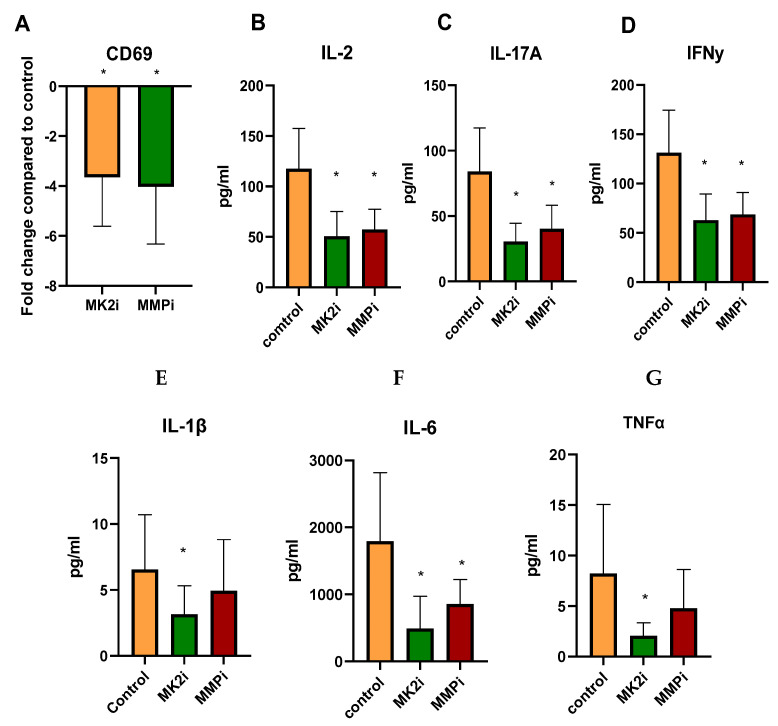
T cell activation markers are increased in active CD tissues but decreased when incubated with MK2i and MMPi where (**A**) CD69 gene expression is decreased in samples incubated with MK2i and MMPi compared to vehicle control and (**B**) IL-2, (**C**) IL-17A, (**D**) IFNγ, (**E**) IL-1β, (**F**) IL-6, and (**G**) TNFα are decreased in tissue supernatants incubated with inhibitors. N = 8, * *p* < 0.05.

In order to confirm the findings with Jurkat cells, indicating that MMPs cleave checkpoint molecules and increase activation and cytokine production, human 4 mg CD tissues were incubated with vehicle control, MK2i, or MMPi for 18 h. Tissues were examined for CD69 gene expression and MK2i was found to decrease expression by 3.64-fold, and MMPi similarly decreased expression by 4.03-fold, suggesting that the inhibitors may have an impact on T cell activation in tissue (Figure 7A). Furthermore, when supernatants were examined for cytokine production, IL-2 was significantly decreased as a marker of T cell activity (Figure 7B), and IL-17A and IFNγ were also decreased as markers of pathologic T cells in CD (Figure 7C,D). The known MK2 downstream cytokines IL-1β, IL-6, and TNFα, which also may be pathogenic in CD, were also significantly decreased with MK2i, but only IL-6 was decreased with MMPi (Figure 7E–G). Thus, confirming in vitro studies, T cell activity and inflammatory cytokine production were decreased ex vivo in CD tissues with inhibitors, suggesting that MK2i has therapeutic potential for CD.

## 3. Discussion

Although CD and UC are both diseases of chronic inflammation, there continue to be questions about differences in these two diseases that are puzzling. One difference in the immune response between the two diseases may be in the T cell responses, with CD having a stronger Th1 signature with IFNγ, but Th17 are also thought to play a role [22]. T cell responses in general are regulated by checkpoint molecules. Checkpoint molecules are in place to maintain tolerance, which is particularly important in the GI tract because of the vast microbial community. However, in CD, there is a loss of tolerance and an overactive T cell response, which leads to pathologies. Thus, dysregulation of checkpoint molecules is one area that needs further examination in order to understand IBD inflammatory pathologies.

The most well-studied checkpoint molecule expressed on antigen presenting cells and other cell types, such as fibroblasts, is PD-L1 [23]. Studies have shown that dysregulation of expression of PD-L1 promotes hyperactive T cell responses in IBD [9,24,25]. In previous work, we showed that PD-L1 expression by fibroblasts in CD could be cleaved by MMPs [10]. Here, we confirmed that this may also occur on monocytes/macrophages by examining the THP-1 cell line. Our work is in agreement with a study by Dezutter-Dambuyant et al. that showed MMP cleavage of PD-L1 by mesenchymal stromal cells. However, this study also indicated that PD-L2 was regulated by MMP9 and MMP13. We found soluble PD-L2 in CD tissue supernatants but did not examine this further because PD-L2 appeared to be regulated differently than PD-L1 in our system, in a non-MK2-dependent manner.

Checkpoint molecules expressed by T cells are also critical in regulating T cell activation, proliferation, and function. Lag3 is known to bind to class II MHC and suppress T cell function [4]. Early studies of Lag3 suggested that Lag3 inhibited T cell activation and that blocking Lag3 with antibodies led to increased CD69 expression and the production of Th1-associated cytokines [26]. Our study is in agreement with this study where we showed that, after incubation with MMPs, Lag3 was cleaved into supernatants and expression of CD69 and production of IL-2 and IFNγ were increased by Jurkat cells. Soluble Lag3 has also been shown in cancer studies to activate antigen-presenting cells [27]. Thus, there is potential that soluble Lag3 released from CD tissues could go on to exacerbate inflammation by activating macrophages and other APCs and thus promote further inflammation in IBD. Lag3 has also been suggested to be expressed by regulatory T cells and able to suppress gut macrophages and mucosal T cells in colitis [6], further suggesting that the cleavage of Lag3 in IBD is detrimental. BTLA is the other checkpoint molecule expressed by T cells that we investigated here. Less is known about the impact of the cleavage of this molecule from CD tissues; however, it has been suggested that is a critical molecule in protecting against mucosal damage in IBD [8]. In sepsis, soluble BTLA has been associated with disease severity [28], thus, more studies are needed to understand the impact of this molecule in IBD. 

We have been examining the MK2 pathway for its role in inflammation and colitis-associated cancer [18]. Previous work by us and others has shown that MK2 regulated cytokine production implicated in CD inflammation, such as IL-1β, IL-6, and TNFα [17,18,19]. Here, we also show in ex vivo studies that treatment of tissue with MK2i leads to decreased IL-1β, IL-6, and TNFα, which is in line with our previous work as they are known to be involved in chronic inflammation in IBD. However, we also found a previously unrecognized role for MK2 in regulating MMP expression. We found that incubating CD tissues with MK2 inhibitors led to decreased MMP1, MMP2, MMP10, and MMP12, but other MMPs were not significantly changed. We also found this regulation to be at the gene level where tissues incubated with MK2 inhibitors had decreased MMP gene expression. Although this is the first study to suggest that MK2 may directly regulate expression of some MMPs, one group demonstrated that MK2 inhibition attenuated MMP2-dependent cancer cell migration [29]. Another group further confirmed the association of MK2 with MMP2 by transfecting cancer cells with constitutively active MK2, demonstrating that this was associated with high MMP2 activity [30], and one more study also suggested that MK2 is associated with MMP2 and MMP9 activity in bladder cancer [31]. However, to our knowledge this is the first study to suggest that MK2 is associated with MMP activity in CD. 

The consequences of checkpoint cleavage were investigated by examining macrophage and T cell responses by cytokine production and the activation marker CD69. In culture with MMP inhibitors, THP-1 cells showed decreased PD-L1 cleavage, and Jurkat cells showed decreased BTLA and Lag3 cleavage. Furthermore, Jurkat cells also showed decreased CD69 expression and decreased production of IL-2 as T cell activation markers, and IFNγ indicative of a Th1 response. These responses are an important part of the pathogenesis seen in CD by contributing to the elevated levels of inflammatory cytokine production in active disease that disrupt barrier function. A limitation of the study is that cell lines had to be used as a tool to model T cell responses in CD. However, in our previous work, CD4^+^ T cells from patient donors were examined, suggesting that the cell line model follows that Th1 and Th17 responses from the previous study [10]. We also performed experiments in an ex vivo approach, which may have some limitations and tissue may change over time, but because we were able to see cytokine changes that mimicked cell culture changes, the two approaches complement one another.

Our previous work also showed that MMP inhibition led to decreased Th1 and Th17 development from naïve CD4 T cells in primary cultures with fibroblasts from CD patients [10]. Furthermore, in ex vivo experiments, we showed that incubation of CD tissues with MK2i or MMPi have decreased T cell activity through CD69 expression and cytokine production. Thus, taken together, MMP inhibition has the potential to limit inflammation in CD, and overall MMPs have clearly been shown to promote pathogenesis in IBD [32,33]. However, drugs targeting MMPs have not shown much progress in clinical trials [13,34]. Despite those findings, MK2 inhibition may be a mor effective treatment approach because MK2 regulates MMP production and cytokine production. Here, we present evidence that MK2 regulates specific MMPs in CD and show that the cleavage of checkpoint molecules by specific MMPs (1, 2, 10, and 12) increases T cell activation and Th1-associated cytokine production. Given our data and the published information on MK2 regulation of inflammatory cytokine production and MMP expression, and the safety information on MK2 inhibitors in human trials [35,36], these pharmaceuticals should be considered as a novel therapeutic approach for CD.

## 4. Materials and Methods

### 4.1. Tissue Collection and Processing

CD and UC tissues were collected under IRB-approved protocols at the University of New Mexico (10-513) for discarded surgical resections and 00127500 for the use of biopsy samples from the GI and IBD Tissue Bank at the University of Utah. Samples were collected from patients with no GI pathologies or CD with active disease or in remission. Sample data are provided in a de-identified manner in Table 1. Tissue samples were divided into 4 mg +/− 0.3 mg and incubated in RPMI complete media for 18 h. Some samples were incubated with MK2 inhibitor (PF-3644022, Sigma Aldrich) at 50 μM, the MMP inhibitor GM6001 at 200 μM, or vehicle (DMSO) control for 18 h, and supernatants were collected for multiplex analysis.

### 4.2. Cell Lines

Jurkat and THP-1 cells were obtained from ATCC and maintained in RPMI supplemented with 10% FBS, 1% L-glutamine, and 1% Penicillin/Streptomycin (RPMI 10% complete media).

### 4.3. Immunofluorescence

Biopsy samples were snap frozen in optimal cutting temperature compound (OCT) and sliced to a 10-micron thickness using a cryostat. Sections were fixed with 4% PFA for 15–20 min at room temperature (RT) and left to air dry. After drying, sections were blocked with 2% BSA for 1 h and stained with anti-human MK2 overnight at 4 °C, followed by AF488 for 1 h at RT. Mounting media with DAPI was added to the section, and slides were imaged using an EVOS Auto2 microscope (ThermoFisher Scientific).

### 4.4. Multiplex Arrays

Tissue and cell supernatants were run in Milliplex arrays for soluble immune checkpoint molecules (Human Immune Oncology Panel), cytokines (Human Cytokine Panel 1), and MMPs (Human MMP1 and MMP2 panels) from MilliporeSigma according to the manufacturer’s instructions. Plates were analyzed on a Luminex MagPix instrument.

### 4.5. Quantitative Real Time PCR

Tissue pieces were homogenized in TRIzol^TM^ reagent (cat. 15596026, ThermoFisher Scientific, Waltham, MA, USA), and RNA extraction was performed according to the manufacturer’s instructions. The quality and quantity of RNA were measured with a NanoDrop^TM^ Lite Spectrophotometer (ThermoFisher Scientific, Waltham, MA, USA). Total RNA (100 ng/µL) was reverse transcribed using High-Capacity cDNA Reverse Transcription Kit (cat. 4368814, ThermoFisher Scientific, Waltham, MA, USA) with the following PCR settings: 25 °C for 10 min, 37 °C for 120 min, and 85°C for 5 min. Quantitation of mRNA was performed using real-time PCR with validated FAM dye-labeled TaqMan^®^ probes (Applied Biosystems, Foster City, CA, USA) for *ACTB*, *MK2*, *MMP1*, *MMP2*, *MMP10*, *MMP12*, and *CD69*. The reaction mixture consisted of cDNA, TaqMan^®^ Fast Advanced Master Mix (Applied Biosystems, Foster City, CA, USA), TaqMan^®^ Assays, and RNase-free water in a total volume of 10 μL. Cycle parameters for TaqMan^®^ assays were as follows: initial denaturation at 95 °C for 3 min, followed by 40 cycles of sequential incubations at 95 °C for 15 s and 60 °C for 1 min. Results were normalized to the expression of *Actb* gene, i.e., housekeeping gene. All experiments were performed as duplicates on QuantStudio™ 5 Real-Time PCR System (ThermoFisher Scientific, Waltham, MA, USA). The endpoint used in real-time PCR quantification, Ct parameter, was defined as the PCR cycle number that crossed the signal threshold. Quantification of gene expression was performed using the comparative CT method (Sequence Detector User Bulletin 2; Applied Biosystems) and reported as the fold change relative to the mRNA of the mouse housekeeping gene, *ACTB*.

### 4.6. MMP Cleavage Assays

Jurkat cells were stimulated with Cell Stimulation Cocktail (ThermoFisher Scientific) for 48 h in 10% RPMI complete media, while THP-1 cells were stimulated with 1 μg of LPS for 48 h. Cells were then fixed with 2% PFA for 15 min at RT then washed with 1X PBS. Human MMP1, 2, 10, and 12 were purchased from AnaSpec (Fremont, CA, USA) and resuspended in assay buffer containing 50 mM Tris, 10 mM CaCl2, 150 mM NaCL, and 0.05% *w*/*v* Brij-35 (TCNB) at pH 8.0 in a flat-bottom 96-well plate. MMPs were added to the cells to a final concentration of 200 ng/mL, along with 1 mM APMA, and MMPs 1, 2, and 10 were incubated for 3 h, while MMP12 was incubated for 24 h at 37 °C. Following the appropriate incubation period, the cells and TCNB media were centrifuged, and supernatants were collected and stored at −80 °C for further analysis.

### 4.7. Jurkat and THP-1 Co-Culture Assays

A total of 1 × 10^5^ Jurkat and THP-1 cells were added at a 1:1 ratio in RPMI 10% complete media in flat-bottom 96-well plates. Jurkat cells were stained with Cell Trace Violet (CTV) (ThermoFisher Scientific) according to the manufacturer/s instructions. Some cells were added to media containing GM6001, a broad MMP inhibitor (MilliporeSigma), and incubated for 15 min at RT. Cell Stimulation Cocktail (ThermoFisher Scientific) and LPS (Enzo Life Sciences) were then added to cells, and media containing GM6001 and cells were incubated individually and in co-culture conditions at 37 °C for 24 h. Following 24 h incubation, media was collected for multiplex array analysis and cells were stained for CD69-APC clone FN50 (ThermoFisher Scientific) for flow cytometry. Flow cytometry analysis was performed using an Attune^TM^ NxT Flow Cytometer and analyzed with Attune^TM^ NxT Software (ThermoFisher Scientific, Waltham, MA, USA).

### 4.8. Statistical Analysis

Results were expressed as the mean ± SE of data obtained from at least three independent experiments, each performed in triplicate. Differences between means were evaluated by one-way ANOVA for multiple comparisons and Student’s *t*-test for the analysis of the significance between two groups. Values of *p* < 0.05 were considered statistically significant. Association between MK2, MMPs, and cytokines were analyzed using Pearson correlation analysis.

## 5. Conclusions

The MK2 pathway was found to be increased at the gene and phosphorylation level in CD, but not in UC. MK2 was found to regulate MMP1, MMP2, MMP10, and MMP12 at the gene level and subsequently checkpoint molecule cleavage from tissues as indicated by incubating tissues with MK2 inhibitor drug. This cleavage was associated with increased T cell activation, and cytokine production was decreased by MMP inhibitor drug in culture and in tissues. Taken together, our data suggest that MK2 could be a novel therapeutic target for CD.

## Figures and Tables

**Table 1:** Sample number per location.

	Normal	UC	CD
Ileum	6	0	10
AC/transverse	6	7	6
DC/sigmoid	6	6	3
Rectum	5	5	6
Mixed	0	9	5
**Total**	**23**	**27**	**29**

## Data Availability

All data is contained within the article.
